# To what extent does surrounding landscape explain stand-level occurrence of conservation-relevant species in fragmented boreal and hemi-boreal forest?–a systematic review protocol

**DOI:** 10.1186/s13750-022-00287-7

**Published:** 2022-10-15

**Authors:** Malin Undin, Anita Atrena, Fredrik Carlsson, Mattias Edman, Bengt Gunnar Jonsson, Jennie Sandström

**Affiliations:** 1 Department of Natural Sciences, Mid Sweden University, 851 70 Sundsvall, Sweden; 2 Department of Wildlife, Fish and Environmental Science, Swedish University of Agricultural Sciences, 901 83 Umeå, Sweden

**Keywords:** Biodiversity; Continuity; Deadwood-dependent species; Deforestation; Habitat loss; Indicator species; Isolation; Landscape configuration; Red-listed species; Taiga

## Abstract

**Background:**

Silviculture and land-use change has reduced the amount of natural forest worldwide and left what remains confined to isolated fragments or stands. To understand processes governing species occurrence in such stands, much attention has been given to stand-level factors such as size, structure, and deadwood amount. However, the surrounding matrix will directly impact species dispersal and persistence, and the link between the surrounding landscape configuration, composition and history, and stand-level species occurrence has received insufficient attention. Thus, to facilitate optimisation of forest management and species conservation, we propose a review addressing ‘To what extent does surrounding landscape explain stand-level occurrence of conservation-relevant species in fragmented boreal and hemi-boreal forest?’.

**Methods:**

The proposed systematic review will identify and synthesise relevant articles following the CEE guidelines for evidence synthesis and the ROSES standards. A search for peer-reviewed and grey literature will be conducted using four databases, two online search engines, and 36 specialist websites. Identified articles will be screened for eligibility in a two-step process; first on title and abstract, and second on the full text. Screening will be based on predefined eligibility criteria related to a PECO-model; *population* being boreal and hemi-boreal forest, *exposure* being fragmentation, *comparator* being landscapes with alternative composition, configuration, or history, and *outcome* being occurrence (i.e., presence and/or abundance) of conservation-relevant species. All articles that pass the full-text screening will go through study validity assessment and data extraction, and be part of a narrative review. If enough studies prove comparable, quantitative meta-analyses will also be performed. The objective of the narrative review and the meta-analyses will be to address the primary question as well as six secondary questions, and to identify important knowledge gaps.

**Supplementary Information:**

The online version contains supplementary material available at 10.1186/s13750-022-00287-7.

## Background

Logging of trees for forestry and other land-use change is a leading cause of habitat loss worldwide, with negative consequences for many forest associated species [1, 2]. In addition to habitat loss per se, logging tends to fragment the remaining forest into smaller, isolated units (hereafter referred to as stands; 3–5). Species’ response to habitat loss can be amplified, alleviated, or otherwise altered by fragmentation [6–8]. In addition, effects of habitat loss and fragmentation can be difficult to disentangle [9, 10]. Yet, a growing body of evidence suggests that fragmentation of historically continuous, suitable habitats have direct consequences for many conservation-relevant species, including birds, mammals, beetles, fungi, and lichen [6, 9, 11–14, but see 8].

The negative consequences of fragmentation have often been linked to direct or indirect effects of a reduced population size [6, 15, 16]. The most studied aspect explaining small populations in fragmented habitats is poor recruitment and reduced (functional) connectivity caused by long distances between suitable habitat patches [3, 12, 17]. However, the empirical support for this is somewhat inconclusive [17]. Additionally, small populations are prone to local extinction due to stochastic events [16]. Fragmentation can also result in lowered fitness, for instance due to edge effects [12, 18] or loss of geneflow, which in turn results in genetic impoverishment, inbreeding depression, and/or lowered adaptability [15, 19–22]. The latter can be further exaggerated due to adverse environmental conditions (23, 24, e.g. climate change, 25). Adding to this, modelling suggests that climate change itself will aggravate forest fragmentation [26], and that fragmentation, in turn, can change local climate, temperature, and wind conditions, potentially causing further stress to already declining populations [27]. Theoretically, species with lower dispersal ability and a shorter lifespan should be more sensitive to fragmentation [28], and different organism groups and species will therefore be affected by fragmentation at different spatial and temporal scales [6, 29, 30].

The most studied aspect related to forest stand-level diversity is stand size [12, 31–33]. A common approach in these studies is biogeography models that effectively treat fragments as islands [29, 34, 35]. However, compared to actual islands, forest stands fall along a gradient from effectively continuous populations, to functioning meta-populations with a balance of extinction and (re)colonization, to non-viable meta-populations where sub-populations lose connectivity and slowly disappear [36, 37]. The configuration, composition, and history of the surrounding landscape (or matrix) will directly affect where along this gradient a stand sits [29, 38–40]. Despite this, the effect of landscape-level variables on stand-level diversity remains underappreciated [12, 29, 41–46]. For example, Oettel and Lapin [47] reviewed 162 studies conducted in European forests and found that only 16% of biodiversity and management indicators could be directly linked to landscape factors. Instead, the most common indicators were found to relate to stand size, structure, and deadwood amount [47].

However, the theoretical importance of spatial and temporal changes to both landscape context and configuration is well understood [7, 9, 48–51]. Furthermore, the number of empirical studies on the effects of forestry and fragmentation with landscape focus are increasing (from a low starting point; 28, 44) and have resulted in a few reviews. In a review, Andren [6] showed that there seemingly is a threshold value for proportion of remaining habitat for both birds and mammals; below this threshold, landscape configuration becomes more important for species occurrence and abundance than habitat amount. Another review, including 30 studies on saproxylic organisms (mainly beetles) across Europe, found significant effects of the surrounding landscape (1–10 kms outside the stand depending on the study; 13). Still, some reviews addressing forest fragmentation and diversity leave out the landscape aspect entirely [53, 54]. In addition, review articles that do include landscape aspects have generally focused on one particular aspect of landscape, such as connectivity/isolation [55], and/or been restricted to one or a few taxonomic groups, only rarely in combination with geographic restrictions [6, 12, 14, 42, 44, 55, 56]. Lastly, a recent review highlights that remote sensing technology for forest analyses has improved rapidly and generated new opportunities for studies of landscape-level effect [57]. However, the same review found that boreal forest was underrepresented in such studies [57].

The boreal biome contains about 27% of all forest globally [4, 58, 59]. Only about 8.5% of the boreal forest is formally protected [59], which falls way short of the Aichi Biodiversity Target 11 goal of protecting at least 17% of all terrestrial areas by 2020 [60]. The boreal forest has been intensively used for forestry, causing a high level of habitat loss and fragmentation [59, 61, 62]. This fragmentation has resulted in that only about 11% of boreal forest can be classified as ‘intact’, and that the average size of a boreal forest stand is approximately 336 ha [51]. In addition, human impact has been noticeably uneven, resulting in most intact boreal forest being found in Russia and Canada [59, 63]. By contrast, in Fennoscandia, almost 1% of the standing forest has been clear cut annually since the onset of large-scale rotation forestry in the 1950s [4, 61, 64]. In addition, a substantial proportion of the remaining forest has been affected by thinning and/or small-scale harvest [4, 61, 65]. For example, only 2% of the productive, unprotected forest in Sweden can be classified as ‘av naturskogskaraktär’ or ‘pristine-like’ today [66]. Furthermore, even though approximately 65% of Sweden is covered in forest, only 4.6% of the country is covered in > 140 year old boreal forest (one third of which has formal protection; 55, 57).

Taken together, there is an urgent need for sustainable ways to manage remaining natural forests and the species they harbour. In addition, areas with potential for restoration need to be identified, and further damage minimised through improved policies for forestry and land use change [68]. Our proposed systematic review, addressing the question: ‘To what extent does surrounding landscape explain stand-level occurrence of conservation-relevant species in fragmented boreal and hemi-boreal forest? [58, 69–72], has potential to provide stakeholders with information directly relevant for such decision making and policy development. Specifically, we believe that the proposed review will allow stakeholders to better understand the premises for conservation success in fragmented landscapes; interpret post monitoring results (such as reasons for an observed decline in a species); optimise conservation strategy by, for instance, prioritise among interventions, stands, and sites to protect and restore; and plan for green infrastructure (i.e., reconstructed connectivity) in boreal and hemi-boreal forest.

Our proposed review will focus on conservation-relevant species, which we define as any threatened, declining, red-listed, or rare species, as well as those considered to be indicator, flagship, umbrella, or key-stone species, and their relationship to any human-caused difference in the surrounding landscape. Hence, we believe that the proposed review will advance the use of indicator, which are crucial for efficient evaluation of conservation potential and outcomes [47]. It is already known that deadwood-dependent species are vulnerable to lost connectivity and substrata continuity and thus good indicators of pristine like forest [13, 30], but complementary perspectives across species groups are needed. In particular, few of the recognised boreal forest indicator species have defined relationships between landscape-level variables and occurrence on stand-level [47, 56]. Furthermore, the slow turn over, combined with the fact that many species linked to boreal forest are highly specialised and recognised as threatened [73], highlights the urgency to further understand consequences of extinction debt in this zone [13, 28, 30, 45]. Lastly, the boreal zone has large landscape variation due to differences in historical land-use and level of human impact [3, 61, 62, 74], suggesting a high value of meta-analyses.

### Stakeholder engagement

During the development of this protocol, a consultation meeting was held between the review authors and an advisory group consisting of representatives from several Swedish agencies; namely, the Swedish Environmental Protection Agency (SEPA; naturvårdsverket), the Swedish Forest Agency (Skogsstyrelsen), the county administration boards (länsstyrelserna) in Västernorrland, Jämtland, Norrbotten, and Gävleborg, and three of the main forestry companies in Sweden: Sveaskog, Holmen Skog, and SCA.

The meeting was held on the 14th of December 2021. The main objectives of this meeting were to discuss the importance of considering the surrounding landscape in conservation planning and identify knowledge gaps and landscape parameters and other effect modifiers of interest for the stakeholders. This discussion included, among other things, landscape factors of interest, relevant landscape sizes to consider, species groups and forest types of particular interest. The result of this meeting directly affected how we defined our primary and secondary questions, PECO, search terms, eligibility criteria, effect modifiers of interest, and data parameters to extract. This advisory group will be invited again to provide oral or written feedback on the content, readability, and clarity of the forthcoming review before submission.

## Objective of the review

**Fig. 1:**
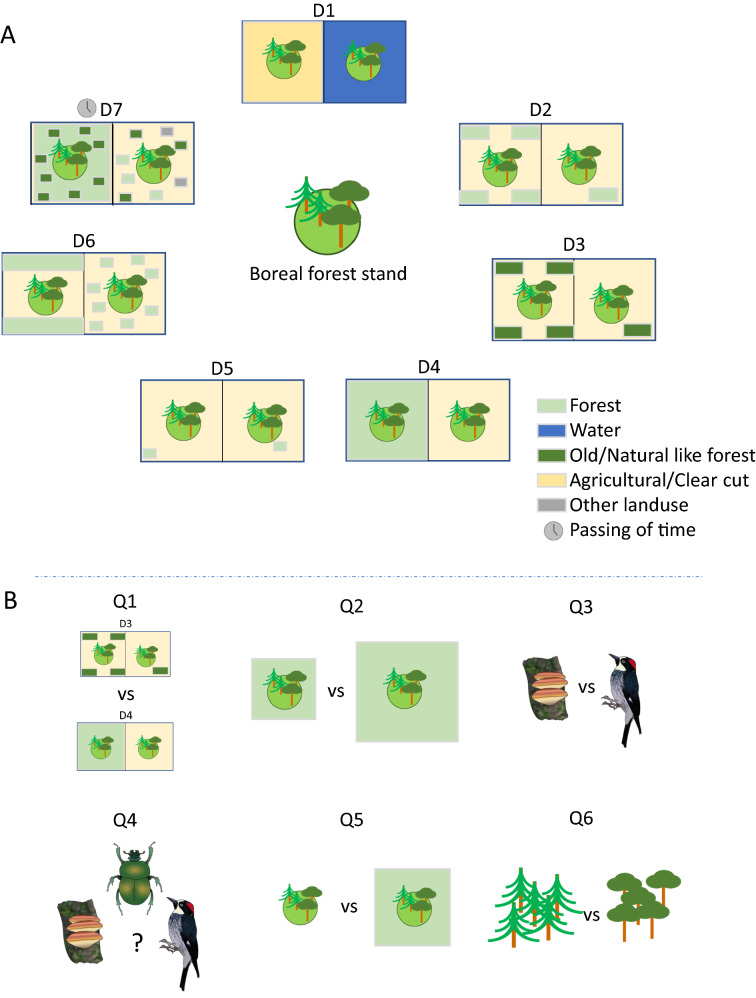
Conceptual illustration of the fact that all forest stands are placed in a landscape context likely to influence the occurrence of conservation-relevant species in said stand. For the proposed review, seven landscape-level variables have been identified relating to the primary question “To what extent does surrounding landscape fragmentation explain stand-level occurrence of conservation-relevant species in boreal and hemi-boreal forest?” **A**. These are: D1 Human fragmentation vs Natural fragmentation; D2 Amount of forest; D3 Amount of old forest; D4 Other matrix composition differences; D5 Distance to forest; D6 Spatial distribution of forest; D7 Intensity/Extent of land use change over time. In addition, six secondary questions have been identified to further break down the primary question. These relate to: Q1 relative effect size of different landscape factors; Q2 the effect of landscape size; Q3 how effects differ between organism groups, Q4 where knowledge gaps remain; Q5 the relative importance of landscape-level factors compared to stand-level factors; Q6 how effects differ between forest types **B**

Given the critical value of the remaining stands of natural boreal forest, the continued forest harvesting and land use change, and the importance of dispersal and landscape permeability for species diversity, it is logical to assume a substantial effect on both incidence and abundance of conservation-relevant species in boreal forest stands. The main objective of the proposed review will be to assess the effects of human caused landscape fragmentation on conservation relevant species. We will achieve this by exhaustively synthesising articles that have investigated the occurrence of one or several conservation-relevant species at forest stand-level, in relation to the seven landscape differences listed under *Comparator* below (Fig. 1A).

The primary question, ‘to what extent does surrounding landscape explain stand-level occurrence of conservation-relevant species in fragmented boreal and hemi-boreal forest?’ can be broken down into the following population, exposure, comparator, outcome (PECO) elements:

*Population*: Boreal and hemi-boreal forest, defined as any forest within the boreal zone and the hemi-boreal transition zone which cover all or parts of the following countries: Canada, Scotland, Iceland, Norway, Sweden, Finland, Estonia, Latvia, Lithuania, Belarus, Russia, Mongolia, Japan, and the American states Alaska, Maine, and Minnesota [58, 69–72].

*Exposure*: Fragmentation and habitat loss, defined as the breaking apart of larger forest tracts into smaller forest stands surrounded by a matrix directly affected by forest harvesting and/or other land-use changes.

*Comparator*: Stands in a landscape that differ in terms of fragmentation, the following seven types of differences compared: D1 Human fragmentation vs Natural fragmentation; D2 Amount of forest in the matrix; D3 Amount of old forest in the matrix; D4 Other matrix composition differences; D5 Distance to forest from stand; D6 Spatial distribution of forest in the matrix; D7 Intensity/Extent of land use change in the matrix over time.

*Outcomes*: Occurrence, defined broadly as incidence, abundance, or composition of conservation-relevant species. Conservation-relevant is defined as species highlighted by the study authors as rare, threatened, red-listed, indicator, keystone, flagship, or umbrella species; locally, regionally, nationally, or globally. In addition, given the key role of dead wood for forest biodiversity, any deadwood-dependent species or species group will be considered conservation-relevant.

In addition to the primary question, we, together with our advisory group, have identified a number of effect modifiers of particular interest for conservation management in the boreal forest (Fig. 1B). Accordingly, we will address the following secondary questions: (1) What type of landscape factors have the strongest effect on stand-level diversity? This question will address the seven comparators listed above and help answer what constitutes the greatest barrier to dispersal and persistence in a fragmented boreal forest landscape. (2) Which landscape size has the strongest explanatory power for stand-level diversity? This question has direct implications for landscape management as well as for evaluating the conservation value of individual forest stands. We expect a bell-shaped relationship where landscapes larger and smaller than a certain size will have lower explanatory power. However, this may be masked when combining studies of organism groups with widely different dispersal abilities. Thus, we ask: (3) Does the surrounding landscape affect organism groups or species differently? This is expected since groups and species differ in, for instance, dispersal ability and longevity. We predict that the greater the dispersal ability and the more general its habitat criteria, the less landscape difference will affect stand-level incidence. On the contrary, organisms with limited dispersal may face a fragmentation threshold already at moderate fragmentation, beyond which further fragmentation makes little difference. Key questions for future research are then: (4) For which organism groups do the largest knowledge gaps remain? Further, (5) What are the relative contributions of stand and landscape factors for stand-level diversity? Lastly, we ask: (6) Does forest type affect the answer to any of the preceding questions? A division among forest types makes sense from a forest management perspective, making this analysis important. If differences between forest types are found, these may be driven by the different species pool associated with different forest types.

## Methods

The proposed review will follow the standards and guidelines from the Collaboration for Environmental Evidence (CEE; [61]) and the reporting standards from ROSES (see Additional file 1) [62]. In addition, this protocol has been registered in PROCEED (https://www.proceedevidence.info/) with the manuscript number PROCEED-22-00027, and the title is publicly available and search able in the online evidence synthesis tool Cadima (version 2.2.3; see further below).

### Search strategy

For the proposed review, we will search for peer-reviewed articles in four bibliographic databases, and two search engines (Table 1). For Web of Science Core Collection, PubMed, SCOPUS, and CAB abstracts (all accessed via subscriptions to the Mid Sweden University Library) we will use the search string specified below adapted to each individual search engine syntax. For google, and google scholar, two sets of simplified versions of the search string will be used and the first 200 hits from each of these four searches will be exported using the online tool Publish or Perish (version 8.2.3944) and further screened. For the four bibliographic databases, no stopping criteria will be used; all identified articles will be screened. In addition, 36 specialist websites will be screened for additional peer-reviewed articles, and grey literature, mainly Master’s theses, PhD theses, and reports (Table 1). These sources were selected based on what has been used for previous reviews that cover the same geographic region and similar topics (e.g., 63).

**Table 1:** Sources to be used during the search for relevant articles

Source type	Source site/organisation	Main country
Data bases	Web of science core collection; SCOPUS; PubMed; CAB abstracts	International
Online search engines	Google Scholar; Google	International
Specialist websites	Environment Canada	Canada
	Natural resources Canada	Canada
	Parks Canada	Canada
	Luke (Natural resources institute of Finland)	Finland
	Metsähallitus (the Finnish forest administration)	Finland
	Ministry of agriculture and forestry in Finland	Finland
	SYKE (Finnish environment institute)	Finland
	Valto (Finnish Ministries’ publications archive)	Finland
	NORA (Natural Environment Research Counsil Open Research Archive)	Great Brittan
	UK Environment Agency	Great Brittan
	bioRxiv (online archive for unpublished preprints in biology)	International
	Conservation Evidence	International
	EU publications	International
	European chapter of the society for ecological restoration (SER)	International
	European commission joint research centre	International
	European environment agency	International
	International union for conservation of nature	International
	Nordic council of ministers	International
	Society for ecological restoration	International
	The international boreal forest research association (IBFRA)	International
	United Nations Environment Programme	International
	BioFokus	Norway
	Landbruksdirektoratet (Norwegian agricultural agency)	Norway
	Miljødirektoratet (Norwegian environment agency)	Norway
	NIBIO (Norwegian institute of bioeconomy research)	Norway
	NINA (Norwegian institute for nature research)	Norway
	Norwegian forest and landscape institute	Norway
	NatureScot (Scotland’s nature agency)	Scotland
	DiVA (Thesis database)	Sweden
	Jordbruksverket (Swedish Ministry of Agriculture)	Sweden
	Länsstyrelser i Sverige (County Administrative Boards in Sweden)	Sweden
	Naturvårdsverket (Swedish Environmental Protection Agency)	Sweden
	Skogsstyrelsen (Swedish Forest Agency)	Sweden
	Svensk fågeltaxering	Sweden
	Uppsök (Thesis database)	Sweden
	United states environmental protection agency	USA
	United states forest service	USA
Supplementary searches	Reference lists of related review articles	n/a

Due to limitations within the review group, searches will be restricted to articles with title and abstract (or equivalent) written in English, Swedish or Norwegian. If and when articles of interest written in a different language are identified based on English title and abstract, assistance with translation will be considered on a case by case basis. We acknowledge that this linguistic bias is a potential shortcoming, but previous reviews on related topics (e.g., 63) makes us confident that searches in these languages will be sufficiently comprehensive. Supplementary searches will be done through the reference lists of related reviews [such as 6, 8, 13, 26, 37, 64, 65]. The reference processor Mendeley (Mendeley Ltd.) will be used to import, collate, and convert references to allow importation to the online evidence synthesis tool Cadima (version 2.2.3). Cadima will then be used to remove duplicates as well as for evaluation and screening of the search results (see below). An annual search update will be performed until the proposed review is published. No restrictions regarding time of articles will be used.

#### Search string and comprehensiveness of the search

The search terms for the proposed review were identified through a combination of reviewing relevant articles, brainstorming within the team, discussion during the stakeholder meeting, and searches in synonym databases. We initially identified 26 benchmark articles (Additional file 2) which were used to refine and test the comprehensiveness of the search string. As long as not all benchmark articles were found, the search terms were adapted accordingly (Additional file 3). Web of Science Core Collection (WoS) was used during this process, and accordingly, only articles indexed in this database were included on the benchmark list. When a search string that detected all 26 articles had been identified, 17 additional (total 43; Additional file 2) articles were added to the benchmark list to confirm the comprehensiveness of the search string and reduce risk of bias. The additional benchmark articles were identified through a combination of being already known to the research group, searches through reference lists of known articles, and independent searches through Google Scholar. The latter served to ensure that the benchmark articles were widespread both geographically and in terms of focal species.

After the extension of the list of benchmark articles, only minor changes were done before the final search string was identified (Additional file 3; Table S2). The final search string found all 43 benchmark articles, and consisted of four blocks (Table 2). For the actual search, all blocks will be combined using the Boolean operator AND. Terms within each block will be combined using the Boolean operator OR unless otherwise specified (Table 2).

**Table 2:** Final search string with four blocks formatted for Web of Science Core Collection

Block	Terms
1. Population	ALL = ((forest* OR wood* OR deadwood* OR dead-wood*) AND (boreal* OR boreonemoral OR hemiboreal OR hemi-boreal OR taiga OR Sweden OR Finland OR Fennoscandia OR Norway OR Canada OR Alaska OR Estonia OR Russia OR Scotland OR Iceland OR Mongolia OR Japan OR Siberia OR Latvia OR Lithuania OR Maine OR Minnesota OR Belarus)) AND
2. Exposure/Comparator – Landscape scale	TS = (landscape* OR region* OR spatial OR provinc* OR ‘‘large-scale’’ OR surrounding OR fragment* OR matrix) AND
3. Exposure/Comparator – Fragmentation	TS = (fragment* OR continu* OR connectivity OR isolate* OR ‘‘habitat loss’’ OR woodlot* OR ‘‘forest stand*’’ OR metapopulation OR ‘‘habitat patch*’’ OR configuration OR ‘‘old-growth forest*’’ OR ‘‘woodland key habitat*’’ OR ‘‘management histor*’’ OR ‘‘land-use histor*’’ OR ‘‘land use histor*‘‘ OR ’’historic* ‘‘land use’’) AND
4. Outcomes	TS = (biodiversity OR ‘‘species richness’’ OR distribution OR abundan* OR occurrence OR composition OR extinction* OR diversity OR densit* OR cover OR coloni*ation* OR occupancy OR dispersal OR community OR viab* OR ‘‘population trend*’’ OR activity OR ‘‘species turnover’’ OR nesting OR incidence OR ‘‘genetic diversity’’ OR ‘‘genetic structur*’’ OR ‘‘isolation by distance’’ OR ‘‘isolation-by-distance’’)

The first block defines the relevant population and thus includes terms such as ‘boreal’ and ‘hemi-boreal’ as well as the names of the relevant countries and regions that host these bioregions (Table 2). In addition to the terms ‘forest*’ and ‘wood*’, the term ‘deadwood’ will be used to ensure the inclusion of studies of this crucial resource in boreal forest (30, 61, 68). The desired population terms were not always mentioned in the title or abstract, thus, to ensure the comprehensiveness of the search, the terms in this block will be searched for in all fields (‘ALL = ’ in WoS; Additional file 3).

The two following blocks define the relevant exposure and comparators; block two serves to identify articles with a landscape component, and block three to identify studies of forest fragmentation. The terms ‘forestry’ and ‘logging’ were removed from the search string during the testing process to reduce the number of irrelevant studies directly related to silviculture. The fourth block defines the relevant outcomes, specifically the relevant units of measure for the occurrence of conservation-relevant species. Block two, three, and four all target the ‘topics’ of the articles (‘TS = ’ in WoS), this means that the terms are searched for in the title, the abstract, and the keywords identified by the study authors as well as by WoS. The truncation symbol * is used as a wildcard character, and denotes any number of and combination of characters.

During the development of this search string, a fifth block was trailed that aimed at identifying only studies of conservation-relevant species. However, this block was found to be too restrictive and was removed (Additional file 3). Instead the issue of conservation-relevance will be included as a core part of the eligibility criteria (see below).

Simplified versions of the search string will be used for websites and search engines that do not implement Boolean search operators.

### Article screening and study eligibility criteria

#### Screening process

The online tool Cadima will be used for screening identified articles for relevance. This screening will be conducted as a two-step process; for step one, screening will be based on title and abstract, for step two, screening will be based on the full text. Before the start of each step, the built-in consistency check tool in Cadima will be used. Before step one, consistency check will include 100 titles and abstracts which will be screened by four members of the review team. Cadima automatically provides a kappa value [80]. Any kappa value below 0.6 will result in discussion in the entire group around the inconsistencies, to streamline the interpretation of the criteria as well as the abstracts. The consistency check will then be reiterated until a kappa value above 0.6 is reached. Once this is reached, Cadima will be set to 5% overlap, meaning that the first 5% of titles and abstracts are screened by two authors. Any inconsistencies during this screening will be discussed in the entire group to further refine interpretation of criteria. If agreement can not be reached, authors will err on the side of caution and include such articles to the full-text step. Once all titles and abstracts are screened, the same consistency procedure will be used for full text, but 10% of the articles will be included.

In both steps, the reviewer will evaluate each articles by answering yes/no/unsure to whether the study/studies (1) took place in boreal or hemi-boreal forest, (2) quantified diversity, (3) examined diversity on stand-level, (4) related diversity to the surrounding landscape, (5) looked at conservation-relevant species, (6) included primary data, (7) related to human caused forest fragmentation (see details on eligibility criteria below). Based on the answers, each article will be labelled as ‘include’, ‘exclude’, or ‘unsure’. During the screening of title and abstract, all articles labelled include or unsure will make it to full text screening and reviewers will tend towards inclusion. All articles labelled unsure after full text screening will be checked by two other reviewers, and if uncertainty remains, the articles will be discussed by the full review team. Reviewers will not evaluate articles on which they are listed as co-authors. A record stating the reason for exclusion will be kept for articles excluded during the full text screening and this record will be provided as an additional file to the proposed review. In addition, the standardised flowchart template from ROSES will be used to record the number of articles /studies that pass each step as a record of the sensitivity and specificity of the search and screening. This flowchart will also be provided in the published review.

#### Eligibility criteria and reasons for exclusion

Relevant population. Articles will be included if studies have been conducted in boreal forest or forest in the hemi-boreal (also sometimes referred as boreonemoral) transition zone [58, 69–72]. Consequently, studies from the following countries: Canada, Scotland, Iceland, Norway, Sweden, Finland, Estonia, Latvia, Lithuania, Belarus, Russia, Mongolia, Japan, and the American states Alaska, Maine, and Minnesota, will be included if the study location falls within the boreal or hemi-boreal zone at the discretion of the review authors, i.e., studies will be included or excluded primarily based on where they took place, rather than based on the classification by the study author. For instance, the authors of some studies conducted in the hemi-boreal forest may have used the term temperate forest. For what qualifies as forest, we use FAO’s definition as “land spanning more than 0.5 hectares with trees higher than 5 m and a canopy cover of more than 10 percent, or trees able to reach these thresholds in situ” [81]. The studied forest stands can be managed or unmanaged forest of any age (including clear-cuts), and hence does not have to contain acknowledged high conservation values. Studies looking at urban parks will be excluded.

*Relevant exposure*. For articles to be included, the surrounding forest landscape must have been fragmented through direct human impact, i.e., through the felling of trees for forestry and other land-use change. Articles will be included regardless of whether fragmentation has been followed by replanting and other forestry activities, or land-use changes such as transformation of forest into agricultural land or urban environments. The level of fragmentation in the surrounding landscape can be expressed or defined using a variety of different units, which can be both qualitative categories (such as ‘more’ or ‘less’) and quantitative estimates with numerical values. Studies must have looked at direct effects of fragmentation at landscape scale and thus will be excluded if they have only quantified indirect effects such as, for example, edge effects. Studies looking only at natural fragmentation will be excluded. However, studies using naturally fragmented landscapes as comparators to landscapes fragmented by human activity will be included (see below).

*Relevant comparators and types of studies*. Included studies must have compared two or more landscapes and looked at how landscape context, composition, configuration, and/or historic change affect the occurrence of conservation-relevant species on stand level. We deem any factor describing the area surrounding the stand (also referred to as the matrix) as a landscape factor, and will split studies into the following nine types based on the landscape variable in focus: D1 Human fragmentation vs Natural fragmentation; D2 Amount of forest; D3 Amount of old forest; D4 Other matrix composition differences (such as the occurrence of a particular species); D5 Distance to forest; D6 Spatial distribution of forest; D7 Intensity/Extent of land use change over time. We will not define any maximum or minimum size of the stand, matrix, or landscape (see study validity assessment), instead we will consider the size of the studied landscape an effect modifier. We will include study designs that compare landscapes before and after exposure (BA studies), an exposed landscape to a control (CE studies), landscapes along a gradient (G studies), and multiple levels of fragmentation (such as studies of ‘low’, ‘medium’, and ‘high’ fragmentation; M studies). Studies that compare forest fragments to naturally fragmented landscapes will be considered a special case of CE studies. Examples of what we consider naturally fragmented landscapes are those fragmented by fire, flooding, or storm felling, or those where forest stands occur interspersed in wetland or on small islands. Studies must have examined defined stands or plots within stands, i.e., studies looking at species occurrence only on landscape scale will be excluded. Studies only addressing stand-level factors (such as stand size) and studies looking at where certain conservation-relevant species occur within a single landscape will be excluded. Only primary studies will be included; reviews, meta-analyses, synthesis, policy discussion, simulation, and other types of theoretical modelling will be excluded.

*Relevant outcomes*: Studies of conservation-relevant species of all organism groups will be included. Conservation-relevance includes, but is not limited to, threatened, declining, red-listed, rare, indicator, flagship, umbrella, and key-stone species, and the review authors will directly rely on how this has been described, defined, and delimited by the study authors. Invasive or pest species will not be considered conservation-relevant and studies of such species will be excluded. In addition, the review authors deem all non-invasive, non-pest deadwood-dependent species as conservation-relevant [30, 68]. However, studies only comparing the amount of deadwood per se will be excluded. Studies of any life stage of conservation-relevant species will be included. Relevant measurements of outcomes are those directly related to the occurrence of conservation-relevant species in the studied stands. These measures include, but are not limited to species presence-absence, cover, abundance, density, and vitality. Studies may have conducted direct surveys of individuals, or indirect surveys of, for instance, nests, spores, or genetic material. Preferably, results should be presented on individual species level. However, diversity indexes such as species richness, community composition, or other indexes of diversity will be considered relevant if compiled of only conservation-relevant species or in a way that these can be separated. When indexes are the only reported outcome, study authors may be contacted to access raw data from which the indexes have been calculated.

### Study validity assessment

All individual studies from all articles remaining after full text screening will be subject to study validity assessment (critical appraisal) to reduce the risk of drawing biased, or in other ways misleading, conclusions. This assessment will categorise the studies as being of low, medium, or high risk of bias. This categorising will be based on ten questions which have been chosen based on previous review protocols, on the critical appraisal tool under development by CEE (Version 0.3 Prototype; 63), and a pilot assessment utilising seven of the benchmark articles. These questions relate to the matching of compared stand and landscapes; the accounting for effect modifiers; the method of site selection; the quantification and reporting of variables, the number of replicates in relation to the variance; the accounting for pseudo replication; suitability of outcome parameter, sampling method, and analyses; and lastly the reporting of the outcome (Additional file 4). If the need is identified during the reviewing process, additional criteria and further specification may be developed in an iterative way. Any such changes and their effects of the categorisation of studies will be reported in the final review as deviations from the original protocol.

The overall outcome of the assessment of a study will equal its highest scores for any individual question. I.e., if a study is deemed as having high risk of bias due to insufficient comparability between the analysed landscapes, it will be deemed as having overall high risk, regardless of the remaining factors. If sufficient information is not provided to evaluate a factor, this automatically qualifies as high risk of bias. To ensure consistency, each study will be evaluated by two reviewers, neither of which will be a co-author of the study. When the categorization differs between the two reviewers, these studies will be discussed by the full review team, and if agreement still cannot be reached, the most cautious (highest risk) evaluation will stand. The outcomes of the study validity assessment will be provided as an additional file to the review. This file will include the categorization for each study as well as the reason for all studies deemed as being of high or medium risk of bias. A high risk of bias will not lead to exclusion of a study from the narrative review or meta-analyses but will lead to a more cautious discussion of the results.

### Data extraction

All studies passing full text screening and going through the validity assessment will go through data extraction. Data and meta-data will be extracted in two steps, based on predefined questions. The questions were formulated based on the discussion during the stakeholders meeting, the primary and secondary questions of the review, as well as the effect modifiers identified, and were refined during a pilot extraction of data from seven of the benchmark articles (Additional file 5). To ensure that the results of our review can be accessible for different end users, we will extract all data to a relational database with consistent and documented coding of all variables extracted. This will ensure data integrity, allow flexibility for exploring the evidence base and provide opportunities for targeted queries. The coding options for said database will be finalised in an iterative process once eligibility screening and study validity assessment have been conducted. In step one, all articles will be subject to meta-data extraction for narrative review. During this step, studies from which sufficient data can be extracted, will be labelled as suitable for meta-analyses. If enough studies are found suitable for meta-analyses and comparable to each other to make a biologically meaningful analysis, these will proceed to step two in which effect size and variance will be extracted. Effect sizes of interest include both correlation and mean difference. In some instances, when relevant data is missing, study authors will be contacted to request raw data on species level. Due to resource limitations, data extraction will mainly be done by one of the review authors, but this will be preceded by a consistency check where 10% of articles and studies are extracted by more than one author and any differences discussed and resolved. In addition, the data extraction sheet will be designed and approved by all review authors.

The extracted data and meta-data will include article details (such as title, authors, year, university); location and other effect modifiers (such as country, location, altitude); study design details such as study type (BA, CE, multilevel, gradient, or human caused vs natural fragmentation), comparator type (more forest, more old-forest, closer to forest, spatial distribution of forest, reduction in forest over time, or time since fragmentation), and comparator details (the span of the gradient, time span on historical data included, etc.); and landscape details such as details on the fragmentation and the landscape parameters used (time of fragmentation, amount and type of forest surrounding the stand; Additional file 5). The composition of the studied landscape will be further broken down into the composition of the studied stands and the composition of the surrounding matrix within the studied landscape. For both stand and matrix details on size, forest age, forest composition, vegetation type, productivity, history, naturalness, and configuration will be extracted (Additional file 5). In terms of location, the review authors will extract the coordinates based on maps and simultaneously ensure that the study has been conducted within the boreal or hemi-boreal zone. Information will also be extracted on the study subjects (such as species name, species group, species mobility, and species substrate) and their conservation-relevance (red listed, threatened, indicator species, etc.), and the outcome such as outcome type (occurrence, abundance), the effect size, variance, and direction of the outcome, as well as how this compares to effects of stand-level factors, and the statistical method used (Additional file 5).

Information will be extracted from the text, figure, tables, and (when needed) supplementary material. The image analysing online tool WebPlotDigitizer (version 4.5) will be used when needed to extract numerical values from figures and graphs. When only raw data is provided, the review team will calculate summary statistics. If accurate numbers cannot be extracted from text, figures, or tables, the corresponding study author will be contacted to request original data. When only averages and no quantification of variance is provided (STD, SEM, confidence intervals, etc.), studies will be excluded from the meta-analyses unless raw data can be acquired. Where data are presented from multiple years, these will be combined into a single effect size unless the study authors have stated a clear reason for not doing so.

### Potential effect modifiers/reasons for heterogeneity

A major potential effect modifier is that the distribution of old and relatively untouched boreal forest is not random. In Sweden and Finland, for instance, there is a clear East–West gradient that also coincides with altitude as well as a coast-to-inland gradient [61, 62]. Additional potential effect modifiers we deem need to be considered are listed in Table 3. This list is based on previous reviews and expert knowledge. However, additional effect modifiers may be identified during the screening and data extraction processes, a final list of modifiers will be provided in an additional file to the review.

**Table 3:** List of effect modifiers deemed important for consideration for the proposed review

Category	Effect modifier
Ecological processes	Within and between seasonal variation, i.e., year and timing of study
Landscape factors	Forest cover, age and density matrix type
	Historic land use
	Landscape difference(s) studied
	Landscape country/location and altitude
	Landscape size
	Extent, intensity, and timing of land-use change and forestry
Stand characteristics	Distance to nearest other stand
	Stand density
	Forest type
	Productivity
	Stand size
	Stand age
Methodology	Extent of difference among compared landscapes
	Indirect effects of fragmentation such as edge effects
	Organism group/species studied
	Reason for conservation relevance
	Outcome type evaluated
	Plot size surveyed within stand
	Survey method

### Data synthesis and presentation

All articles going through data and meta-data extraction will be part of a narrative review. During this procedure, individual studies will be deemed as suitable or not for meta-analyses. The aim of the narrative review will be to describe the findings and the quality of the studies, including a summary of the study validity assessment. A narrative synthesis table will be provided in the review and/or as an additional file. So-called vote counting, where the actual number of studies with a certain effect direction is given higher emphasis in the interpretation of the results, will be avoided when summarizing outcomes.

If extraction of effect size and variance is possible from studies for which outcomes that can be combined in a biologically meaningful way, all efforts will be made to complement the narrative review with quantitative meta-analyses. The feasibility of this will be evaluated after narrative data extraction and be based on how many studies have explored comparable effect modifiers, organism groups, and outcomes, as well as on the variance found in the studies, and any interactions between, for instance, organism group and landscape size studied [82]. If meta-analysis is deemed possible, effect sizes will be standardised and weighted appropriately. Effect sizes of interest include both correlation and mean difference. If possible, overall analyses will be complemented with separate analyses of individual organism groups, as well as based on different sizes of landscapes and different forest types to address the secondary question. Furthermore, if possible, studies will be grouped according to different outcome types (incidence, abundance etc.) analysed separately to further break down the landscape-effect. Species level data will be analysed separately from index data (such as species richness). The results of the study validity assessment will be used as a factor in all analyses. Based on the benchmark articles and the pilot data extraction, we identify differences in landscape variable studied, outcome types reported, and description of the stands and matrix as the greatest obstacle for successful and meaningful meta-analysis.

Random effect models will be used when appropriate. Based on pilot searches and extraction, it is likely that many of the relevant studies will have investigated landscapes along one or several gradients rather than being of a CE, or BA study design. If these gradient studies can be standardised, and heterogeneity accounted for, they will be combined using meta-regression. Forest plots will then be produced to visualise the effect size and confidence interval for each included study. Risk of publication bias will be assessed using funnel plots with an associated Egger’s regression (83) utilizing the inverse square root of the sample size as a measure of precision [84]. For additional analyses of robustness, fail-safe numbers, i.e., the number of studies with an average effect size of zero needed to make the combined effect size insignificant, will be calculated [85]. Based on the study validity assessment, the risk of bias will be evaluated through a sensitivity analysis. The strength of evidence for the answers to the primary and secondary questions will be discussed in the light of the number of studies identified, their study validity assessments, and the consistency in direction and size of observed outcomes. Analyses will be conducted in R with the help of the *metafor* package [85], but details around the quantitative analyses will only be known once articles have been screened and data from all studies extracted.

Our key target group for the proposed review is stakeholders directly involved with forest conservation and management. The goal is thus to generate easily interpreted synthesis on what is known about landscape effects on the occurrence of conservation-relevant species in fragmented boreal and hemi-boreal forests. As part of this, we aim to make maximum use of figures and tables as complements to our text, and the review will be accompanied with a number of supplementary materials, including tables of all species included and the geographical location of all studies. Another important aim is to identify knowledge gaps or underrepresented areas, both in terms of landscape factors, organism groups, and landscape size. A number of heat maps cross-tabulating, for instance, landscape size and study species group will be used to identify such gaps. Lastly, to make data accessible the final narrative table and data extraction sheets will be made available online as additional files to the finalised review.

## Supplementary Information

**Additional file 1: **Roses form The filled in ROSES reporting standards form for systematic review protocols.

**Additional file 2: **Benchmark articles. List of the 43 articles used to edit the search string and ensure comprehensiveness of the search.

**Additional file 3: **Search string development. The development of the search string with terms used in the search blocks, how they were combined, how many hits individual blocks as well as block combinations rendered in Web of Science Core Collection, and how many of the benchmark papers could be identified among those hits.

**Additional file 4: **Study validity assessment (critical appraisal) criteria. Table of the criteria and questions that will be used to categorise studies as being of high, medium, or low risk of bias.

**Additional file 5: **Data extraction spreadsheet. Table with the parameters that will be extracted for narrative as well as quantitative review of the identified articles and studies. Included is pilot extraction of seven ofn the benchmark articles.

## Data Availability

Not applicable.
