# Supplementary material for: To what extent does surrounding landscape explain stand-level occurrence of conservation-relevant species in fragmented boreal and hemi-boreal forest?–a systematic review protocol
Source: Environ Evid. 2022 Oct 15;11:32. doi: 10.1186/s13750-022-00287-7 (PMC11378820; doi:10.1186/s13750-022-00287-7)
Supplement: Supplementary file 2 — Additional file 2: Benchmark articles. List of the 43 articles used to edit the search string and ensure comprehensiveness of the search. [file 13750_2022_287_MOESM2_ESM.pdf]

Additional file 2. Benchmark publications used to refine the search string and test its comprehensiveness. The line delimits the initial list of 26 papers from the papers added in the extension-step.

1. Berglund, Håkan, and Bengt Gunnar Jonsson. 2005. "Verifying an Extinction Debt among Lichens and Fungi in Northern Swedish Boreal Forests." *Conservation Biology* 19(2):338–48. doi: 10.1111/j.1523-1739.2005.00550.x.

2. Penttilä, R., M. Lindgren, O. Miettinen, H. Rita, and I. Hanski. 2006. "Consequences of Forest Fragmentation for Polyporous Fungi at Two Spatial Scales." *Oikos* 114(2):225–40. doi: 10.1111/j.2006.0030-1299.14349.x.

3. Sverdrup-Thygeson, A., and D. B. Lindenmayer. 2003. "Ecological Continuity and Assumed Indicator Fungi in Boreal Forest: The Importance of the Landscape Matrix." *Forest Ecology and Management* 174(1–3):353–63. doi: 10.1016/S0378-1127(02)00043-9.

4. Kuusinen, Mikko, and Antti Penttinen. 1999. "Spatial Pattern of the Threatened Epiphytic Bryophyte Neckera Pennata at Two Scales in a Fragmented Boreal Forest." *Ecography* 22(6):729–35. doi: 10.1111/j.1600-0587.1999.tb00522.x.

5. Olsson, Jörgen, Therese Johansson, Bengt Gunnar Jonsson, Joakim Hjältén, Mattias Edman, and Lars Ericson. 2012. "Landscape and Substrate Properties Affect Species Richness and Community Composition of Saproxylic Beetles." *Forest Ecology and Management* 286:108–20. doi: 10.1016/j.foreco.2012.08.033.

6. Ranius, Thomas, Victor Johansson, Martin Schroeder, and Alexandro Caruso. 2015. "Relative Importance of Habitat Characteristics at Multiple Spatial Scales for Wood-Dependent Beetles in Boreal Forest." *Landscape Ecology* 30(10):1931–42. doi: 10.1007/s10980-015-0221-5.

7. Nordén, Jenni, Jens Åström, Torbjörn Josefsson, Stefan Blumentrath, Otso Ovaskainen, Anne Sverdrup-Thygeson, and Björn Nordén. 2018. "At Which Spatial and Temporal Scales Can Fungi Indicate Habitat Connectivity?" *Ecological Indicators* 91(April):138–48. doi: 10.1016/j.ecolind.2018.03.062.

8. Edman, Mattias, Mårten Gustafsson, Jan Stenlid, and Lars Ericson. 2004. "Abundance and Viability of Fungal Spores along a Forestry Gradient - Responses to Habitat Loss and Isolation?" *Oikos* 104(1):35–42. doi: 10.1111/j.0030-1299.2004.12454.x.

9. Brotons, Lluís, Mikko Mönkkönen, Esa Huhta, Ari Nikula, and Ari Rajasärkkä. 2003. "Effects of Landscape Structure and Forest Reserve Location on Old-Growth Forest Bird Species in Northern Finland." *Landscape Ecology* 18(4):377–93. doi: 10.1023/A:1026148825138.

10. Edman, Mattias, Mårten Gustafsson, Jan Stenlid, Bengt Gunnar Jonsson, and Lars Ericson. 2004. "Spore Deposition of Wood-Decaying Fungi: Importance of Landscape Composition." *Ecography* 27(1):103–11. doi: 10.1111/j.0906-7590.2004.03671.x.

11. Komonen, Atte, Reijo Penttilä, Mariko Lindgren, and Ilkka Hanski. 2000. "Forest Fragmentation Truncates a Food Chain Based on an Old-Growth Forest Bracket Fungus." *Oikos* 90(1):119–26. doi: 10.1034/j.1600-0706.2000.900112.x.

12. Dettki, H., and P. A. Esseen. 1998. "Epiphytic Macrolichens in Managed and Natural Forest Landscapes: A Comparison at Two Spatial Scales." *Ecography* 21(6):613–24. doi: 10.1111/j.1600-0587.1998.tb00554.x.

13. Edenius, Lars, and Johan Elmberg. 1996. "Landscape Level Effects of Modern Forestry on Bird Communities in North Swedish Boreal Forests." *Landscape Ecology* 11(6):325–38. doi: 10.1007/BF02447520.

14. Åberg, J., G. Jansson, J. E. Swenson, and P. Angelstam. 1995. "The Effect of Matrix on the Occurrence of Hazel Grouse (*Bonasa bonasia*) in Isolated Habitat Fragments." *Oecologia* 103(3):265–69. doi: 10.1007/BF00328613.

15. Ethier, Kevin, and Lenore Fahrig. 2011. "Positive Effects of Forest Fragmentation, Independent of Forest Amount, on Bat Abundance in Eastern Ontario, Canada." *Landscape Ecology* 26(6):865–76. doi: 10.1007/s10980-011-9614-2.

16. Schmiegelow, Fiona K. A., Craig S. Machtans, and Susan J. Hannnon. 1997. "Are Boreal Birds Resilient to Forest Fragmentation? An Experimental Study of Short-Term Community Responses." *Ecology* 78(6):1914–32. doi: 10.1890/0012-9658(1997)078[1914:ABBRTF]2.0.CO;2.

17. Berg, Åke. 1997. "Diversity and Abundance of Birds in Relation to Forest Fragmentation, Habitat Quality and Heterogeneity." *Bird Study* 44(3):355–66. doi: 10.1080/00063659709461071.

18. Norton, Michael R., Susan J. Hannon, and Fiona K. A. Schmiegelow. 2000. "Fragments Are Not Islands: Patch vs Landscape Perspectives on Songbird Presence and Abundance in a Harvested Boreal Forest." *Ecography* 23(2):209–23. doi: 10.1111/j.1600-0587.2000.tb00277.x.

19. Jokimäki, Jukka, Esa Huhta, Mikko Mönkkönen, and Ari Nikula. 2000. "Temporal Variation of Bird Assemblages in Moderately Fragmented and Less-Fragmented Boreal Forest Landscapes: A Multi-Scale Approach." *Ecoscience* 7(3):256–66. doi: 10.1080/11956860.2000.11682595.

20. Rolstad, Jørund, Magne Sætersdal, Ivar Gjerde, and Ken Olaf Storaunet. 2004. "Wood-Decaying Fungi in Boreal Forest: Are Species Richness and Abundances Influenced by Small-Scale Spatiotemporal Distribution of Dead Wood?" *Biological Conservation* 117(5):539–55. doi: 10.1016/j.biocon.2003.09.008.

21. Virkkala, Raimo, and Ari Rajasärkkä. 2006. "Spatial Variation of Bird Species in Landscapes Dominated by Old-Growth Forests in Northern Boreal Finland." *Biodiversity and Conservation* 15(7):2143–62. doi: 10.1007/s10531-004-6897-5.

22. Snäll, Tord, Anna Hagström, Jörgen Rudolphi, and Håkan Rydin. 2004. "Distribution Pattern of the Epiphyte Neckera Pennata on Three Spatial Scales - Importance of Past Landscape Structure, Connectivity and Local Conditions." *Ecography* 27(6):757–66. doi: 10.1111/j.0906-7590.2004.04026.x.

23. Nordén, Jenni, Philip J. Harrison, Louise Mair, Juha Siitonen, Anders Lundström, Oskar Kindvall, and Tord Snäll. 2020. "Occupancy versus Colonization–Extinction Models for Projecting Population Trends at Different Spatial Scales." *Ecology and Evolution*

10(6):3079–89. doi: 10.1002/ece3.6124.

24. Komonen, Atte, Ilkka Puumala, Gergely Várkonyi, and Reijo Penttilä. 2021. “Wood-Decaying Fungi in Old-Growth Boreal Forest Fragments: Extinctions and Colonizations over 20 Years.” *Silva Fennica* 55(1):1–10. doi: 10.14214/sf.10491.

25. Drolet, Bruno, André Desrochers, and Marie Josée Fortin. 1999. “Effects of Landscape Structure on Nesting Songbird Distribution in a Harvested Boreal Forest.” *Condor* 101(3):699–704. doi: 10.2307/1370205.

26. Segers, J. L., and H. G. Broders. 2014. “Interspecific Effects of Forest Fragmentation on Bats.” *Canadian Journal of Zoology* 92(8):665–73. doi: 10.1139/cjz-2014-0040.

---

27. Johansson, Per, and Johan Ehrlén. 2003. “Influence of Habitat Quantity, Quality and Isolation on the Distribution and Abundance of Two Epiphytic Lichens.” *Journal of Ecology* 91(2):213–21. doi: 10.1046/j.1365-2745.2003.t01-1-00754.x.

28. Gibb, Heloise, Joakim Hjältén, John P. Ball, Ola Atlegrim, Roger B. Pettersson, Jacek Hilszczański, Therese Johansson, and Kjell Danell. 2006. “Effects of Landscape Composition and Substrate Availability on Saproxylic Beetles in Boreal Forests: A Study Using Experimental Logs for Monitoring Assemblages.” *Ecography* 29(2):191–204. doi: 10.1111/j.2006.0906-7590.04372.x.

29. Rukke, B. A., and F. Midtgaard. 1998. “The Importance of Scale and Spatial Variables for the Fungivorous Beetle Bolitophagus Reticulatus (Coleoptera, Tenebrionidae) in a Fragmented Forest Landscape.” *Ecography* 21(6):561–72. doi: 10.1111/j.1600-0587.1998.tb00548.x.

30. Villard, Marc André, M. Kurtis Trzcinski, and Gray Merriam. 1999. “Fragmentation Effects on Forest Birds: Relative Influence of Woodland Cover and Configuration on Landscape Occupancy.” *Conservation Biology* 13(4):774–83. doi: 10.1046/j.1523-1739.1999.98059.x.

31. Gu, W. D., M. Kuusinen, T. Konttinen, and I. Hanski. 2001. “Spatial Pattern in the Occurrence of the Lichen *Lobaria pulmonaria* in Managed and Virgin Boreal Forests.” *Ecography* 24(2):139–50. doi: 10.1034/j.1600-0587.2001.240204.x.

32. Laaksonen, Mervi, Elina Peuhu, Gergely Várkonyi, and Juha Siitonen. 2008. “Effects of Habitat Quality and Landscape Structure on Saproxylic Species Dwelling in Boreal Spruce-Swamp Forests.” *Oikos* 117(7):1098–1110. doi: 10.1111/j.0030-1299.2008.16620.x.

33. Jokimäki, Jukka, and Esa Huhta. 1996. “Effects of Landscape Matrix and Habitat Structure on a Bird Community in Northern Finland: A Multi-Scale Approach.” *Ornis Fennica* 73(3):97–113.

34. Økland, Bjørn, Alf Bakke, Sigmund Hågvar, and Torstein Kvamme. 1996. “What Factors Influence the Diversity of Saproxylic Beetles? A Multiscaled Study from a Spruce Forest in Southern Norway.” *Biodiversity and Conservation* 5(1):75–100. doi: 10.1007/bf00056293.

35. Schroeder, L. Martin, Thomas Ranius, Barbara Ekbom, and Stig Larsson. 2007. "Spatial Occurrence of a Habitat-Tracking Saproxylic Beetle Inhabiting a Managed Forest Landscape." *Ecological Applications* 17(3):900–909. doi: 10.1890/06-0090.

36. Nordén, Jenni, Reijo Penttilä, Juha Siitonen, Erkki Tomppo, and Otso Ovaskainen. 2013. "Specialist Species of Wood-Inhabiting Fungi Struggle While Generalists Thrive in Fragmented Boreal Forests." *Journal of Ecology* 101(3):701–12. doi: 10.1111/1365-2745.12085.

37. Pakkala, Timo, Ilkka Hanski, and Erkki Tomppo. 2002. "Spatial Ecology of the Three-Toed Woodpecker in Managed Forest Landscapes." *Silva Fennica* 36(1):279–88. doi: 10.14214/sf.563.

38. Kouki, J., and A. Väänänen. 2000. "Impoverishment of Resident Old-Growth Forest Bird Assemblages along an Isolation Gradient of Protected Areas in Eastern Finland." *Ornis Fennica* 77(4):145–54.

39. Reunanen, Pasi, Ari Nikula, and Mikko Mönkkönen. 2002. "Regional Landscape Patterns and Distribution of the Siberian Flying Squirrel Pteromys Volans in Northern Finland." *Wildlife Biology* 8(4):267–78. doi: 10.2981/wlb.2002.024.

40. Tjernberg, M., K. Johnsson, and S. G. Nilsson. 1993. "Density Variation and Breeding Success of the Black Woodpecker *Dryocopus martius* in Relation to Forest Fragmentation." *Ornis Fennica* 70(3):155–62.

41. Rolstad, J., and P. Wegge. 1987. "Distribution and Size of Capercaillie Leks in Relation to Old Forest Fragmentation." *Oecologia* 72(3):389–94. doi: 10.1007/BF00377569.

42. Kehler, Daniel, Søren Bondrup-Nielsen, and Soren Bondrup-Nielsen. 1999. "Effects of Isolation on the Occurrence of a Fungivorous Forest Beetle, Bolitotherus Cornutus, at Different Spatial Scales in Fragmented and Continuous Forests." *Oikos* 84(1):35. doi: 10.2307/3546864.

43. Hottola, Jenni, and Juha Siitonen. 2008. "Significance of Woodland Key Habitats for Polypore Diversity and Red-Listed Species in Boreal Forests." *Biodiversity and Conservation* 17(11):2559–77. doi: 10.1007/s10531-008-9317-4.
